# Arsenical Necrosis

**Published:** 1898-02

**Authors:** Otto E. Inglis

**Affiliations:** Philadelphia


### ARSENICAL NECROSIS.[1]

BY OTTO E. INGLIS, D.D.S., PHILADELPHIA.

VERY little has been written in text-books for students upon the subject of the escape of arsenic trioxide from a cavity of decay upon the gum. A general warning to avoid such escape is alone given. Specific directions as to the treatment of any condition that may arise from carelessness in this direction are searched for in vain. Garretson recommends "repeated syringings, and gives the information that the sequestrum which is formed is seldom considerable." The writer has had the good fortune to be placed where a considerable number of such cases fall into his hands, and

---

[1]Read before the Academy of Stomatology, Philadelphia, November 23, 1897.

while he can gladly agree with Garretson in that the sequestrum is seldom considerable, he feels that the information is vague and not sufficient unto the need of the anxious young practitioner or student who has such a case on hand, as the result of his lack of care or ignorance. Accidents will happen, and we should be provided with the antidote,—viz., a knowledge of how to proceed to neutralize or cure their effects.

First, let us consider how arsenic acts upon gum tissue. To this end the scientific experiments of Dr. J. H. McQuillen are worthy of attention. He took a frog, and to the web of its foot applied a grain of arsenic. The effect was noted by the use of the microscope. Dr. McQuillen says,[1] "For the first half-hour or so no apparent change was observable in the circulation: in a short time after this, however, the vessels sensibly increased in size and the circulation in rapidity of movement. The oval-shaped red corpuscles of the frog could be seen not only rushing through the vessels much more rapidly, but also in greater numbers than in the normal circulation. This continued for some time, and then the rapid motion of the circulation gradually decreased, the blood-corpuscles moving along slower and slower and apparently clinging to the walls of the vessels as if unwilling to leave them, and when doing so only yielding on account of the *vis a tergo.* The circulation went on decreasing until eventually complete stagnation supervened. This condition was not confined to the immediate vicinity of the spot on which arsenous acid was placed, but extended over the entire web. . . . The first frog died promptly, and arsenic was found in the muscular tissue of its heart. A second frog, which was treated in precisely the same manner, exhibited similar local inflammatory effects, but the inflamed part sloughed out, leaving an aperture in the web. It lived over a week in the apparent enjoyment of health, and then, through the carelessness of a domestic, it escaped." Dr. McQuillen inferred that arsenic could be classed as an irritant poison. We may infer from these experiments that arsenic is absorbed into the general circulation or prevented from so doing according to the degree of inflammation existing between it and the heart. We have further evidence of this in the application of arsenic for the removal of tumors. If a small quantity be applied systemic poisoning may take place, but when a large quantity is used, stasis is brought about so rapidly between the arsenic and the general circulation that absorption ceases because there is

---

[1] Dental Cosmos, vol. iii. p. 581.

no circulation to superintend absorption. Bearing upon the consideration of this subject is the fact that, according to the authorities, the injection of arsenic produces enteritis. There seems to be no direct evidence that arsenic is a chemical irritant, escharing after the manner of phenol, which combines directly with the albumen of the tissues, although the statement has been made that arsenite of albumen is formed. In an attempt to determine this point the following experiments were performed. With a teaspoonful of fresh egg albumen, ten grains of arsenic trioxide were thoroughly triturated in a mortar at a temperature of 98° F. No coagulation took place at any time during a week or more, while the arsenic gradually settled to the bottom of the mortar, leaving clear albumen supernatant. A high temperature would undoubtedly have coagulated the albumen. A second experiment was made, the arsenic being ground into the albumen and then three times the volume of water added and the mixture again triturated. A magma composed of arsenic and albumen settled to the bottom of the mortar. The question now being whether the coagulum was due to the arsenic or to the water, a third experiment was performed. This consisted in taking one part egg albumen and three parts water and triturating. A flocculent coagulum was at once formed which, lacking the weight of the arsenic, did not settle.

We can only conclude that the coagulum formed by the water held the arsenic suspended in its meshes. The experiments are offered for what they are worth So far as seen arsenic must be classed with irritants which act through the inflammation they are capable of producing.

Biddle[1] states, upon the authority of Scolosuboff, that in arsenical poisoning, if in fresh muscle one part of arsenic be found, the proportion in liver is ten and eight-tenths parts, brain, thirty-six and five-tenths parts, and in the spinal cord thirty-seven and three-tenths parts.

This would seem to show an enormous selective affinity of the drug for nervous tissue. May we not justly infer that this selective affinity accounts for the hyperæmia of parts distant from the seat of application (as in the web of the frog's foot). In other words, that the drug after absorption exhibits an affinity for the vasomotor system producing paralysis of the vasoconstrictor nerves. This being permanent, the capillaries are unable to contract and complete stasis results.

---

[1] Materia Medica and Therapeutics, p. 472.

When arsenic escapes upon a gum, the first effect is probably the irritation of the vasomotor nerves and their subsequent paralyzation. This causes atony of the vessels and in consequence stagnation of blood ensues as may be seen by the turgid condition of the gum festoon.

Later, this festoon becomes of a dirty yellow color, which signifies that it is gangrenous. The extent and direction of the necrosis depend upon the amount of arsenic which has leaked upon the gum. As the amount is generally small, systemic poisoning cannot usually result, although it is probable that some arsenic is absorbed into the general circulation. When leakage of arsenic takes place from a tooth-cavity, different parts are attacked, according to whether the arsenic is confined against the gum septum or allowed to diffuse itself.

In the writer's experience, the mixture of arsenic and creosote, as furnished in the ordinary "nerve paste," has produced more serious results than the pastes in which the arsenic is distended with morphine or other admixture. This is another evidence of the fact that effect is in proportion to the quantity of arsenic allowed to escape.

The following may be held as expressing the amount of damage done:

1. Necrosis of gum septum.
2. Necrosis of gum border encircling one or more teeth.
3. Limited necrosis of bone directly underlying these parts.
4. Extension of the area of necrosis along the pericementum, with loosening and bloodless loss of tooth.
5. Necrosis beginning at some high portion of the alveolus, as when arsenic is applied through a perforation.
6. Necrosis beginning at the apical foramen owing to the forcing through of arsenic.
7. Necrosis of tissues of the cheek or lip.

Let us consider these in their proper order.

### NECROSIS OF THE GUM SEPTUM.

This limited amount of necrosis could of course only occur as the result of leakage of a very small amount of arsenic, or as limited by prompt surgical interference. It will be evidenced in its first stage by a turgid condition of the gum between the teeth. This will be succeeded by a yellow slough. The part should be thoroughly syringed to remove any arsenic present. The tooth-cavity may be opened, the pulp removed, and the canals filled.

The slough should then be curetted away with a large spoon excavator or small lancet. Antiseptic gauze is then to be placed against the gum between the teeth, and the patient is to be directed to syringe the part with three per cent. pyrozone or one to two hundred solution of potassium permanganate in water. Regeneration may be expected.

### NECROSIS OF GUM BORDER ENCIRCLING ONE OR MORE TEETH.

This condition occurs as the result of leakage of a considerable amount of paste, the same not being confined to the dental interspace. It is apt to occur when very fluid preparations have been used in undue quantity. It is peculiar to fluids to follow the festoon of the gum. This may be noted when, during excavating, the gum has been made to bleed. The blood will trickle backward, following the line of the festoons. The same will also be noted with carbolic acid; if applied in excess, the festoons will be eschared. As carbolic acid is a common menstruum for arsenical pastes, it is not necessary to say more on this point.

This would seem to be an especially dangerous manner for the arsenic to distribute itself, as indeed it is, especially in the inferior jaw, where gravitation favors its retention within the gum festoon. The pericementum having its attachment at the festoon is readily attacked and the tooth lost. In the superior jaw the danger is seemingly not so great. At any rate the same disastrous results have not been observed. The dead tissue should be curetted away, the part kept aseptic, and regeneration will usually take place.

### NECROSIS OF BONE DIRECTLY UNDERLYING THE SOFT PARTS.

When a limited amount of leakage has caused necrosis and the same is neglected, a small portion of the alveolar process becomes necrosed and forms a sequestrum. This may persist in its position for many months after separation, owing to the peculiar retentive shape of the bone. A certain amount of liquefaction into pus may result, but, as a rule, in from three to six weeks operative procedures are necessary. The sequestrum should be divided at its middle portion and removed in two pieces.

This death of bone is due probably to the loss of its source of nutrition,—viz., the periosteum, and possibly also to the entrance of organisms to the part. These produce a septic gangrenous condition against which the vital tissues react. The result is demarcation of the bone and its sequestration by a line of pus.

When the bone has been lost, it is found that the adjoining teeth are denuded, their pericementum to an extent nearly corresponding to the size of the sequestrum. Some regeneration will take place, but there will also be more or less deformity resulting from the loss of bone tissue. It has been the experience that when a quantity of arsenic has been used larger than will produce a limited death of bone the pericementum is so affected that the loss of at least one tooth results. When the process has been affected, as will be indicated by its insensitivity at points underlying sloughing gum tissue, it should be gently scraped or burred away until sensitive tissue is reached. The part should be frequently syringed with pyrozone and the result awaited. If nothing else be accomplished by this treatment, the arsenic at least will be removed and the extent of necrosis, in all probability, limited. The following is a description of the case which resulted in the largest sequestrum of process without loss of the tooth that the writer has seen. The application was made to a mesio-occlusal cavity of a molar in January, 1897. In about three weeks a sequestrum of the septum about one-third inch deep was taken away. The lingual and buccal margins of the process were affected about alike.

The case was seen again, November 1, 1897. The gum had receded, exposing the cementum from the middle of the cervix on the distal root to a point about one-quarter inch below the cervix on the mesial root.

The tooth was somewhat looser than normal, due probably to chronic peridental irritation with food fermentation, calculus, etc., as cause. The gum edge was somewhat thickened.

Dr. J. H. McQuillen[1] instances a case in which by syringing with tepid water and applications of creosote and iodine he succeeded in saving two bicuspids, but was compelled to remove a considerable portion of the process, in consequence of which there was considerable disfigurement.

EXTENSION OF THE AREA OF NECROSIS ALONG THE PERICEMENTUM, WITH LOOSENING AND BLOODLESS LOSS OF THE TOOTH.

This may result from confinement of arsenic against the septum or from its distribution around the festoon. These are the cases in which accrue results causing much chagrin to the operator. Nothing can be more disastrous to one's reputation than to bring about a deformity where a simple remedial measure was intended. When the arsenic begins its action upon the pericementum at the gum

[1] Dental Cosmos, vol. xiii. p. 394.

line, whether between the teeth or at the festoon, and the case is neglected for a few days, it may rapidly destroy the membrane. The tooth becomes loose and sore and may in some cases be extracted with the fingers, or even in the attempt to remove a temporary filling.

The surrounding gum tissue is not always as much affected as one would expect. After the tooth is removed the alveolus is found bare, bloodless, and septic. There is no doubt of its septic condition, as the odor of the tooth will resemble that of a tooth extracted for phagedenic pericementitis. The fermentation of stagnant saliva and serum will account for this. Were the case neglected at this point, it is probable that the alveolar cortex or process enveloping the tooth will be thrown off. A specimen is presented, taken from the mouth of a boy of fourteen years. Arsenic was applied to a distal cavity in a superior first bicuspid. The gum septum necrosed. Free curetting was indulged in, but the pericementum had been so profoundly affected that the tooth loosened and was extracted. Later the envelope of process was taken away.

Later, Dr. J. Foster Flagg[1] mentions a case in which a second superior bicuspid and first molar were lost, together with all the intervening process, as the result of a persistent application of arsenic to a distal cavity in the bicuspid. Leakage occurred.

Another phase of the condition was presented in the case of a young girl fifteen years of age. The application was made to a left inferior second molar. The pericementum necrosed, and the tooth was extracted without the loss of a single drop of blood. The apices of the alveoli were drilled away to healthy bone, in order to avoid any possibility of extension of the necrosis in the direction of the inferior dental canal. The writer then decided to experiment upon the case, and directed the patient to make application of three per cent. pyrozone upon cotton three times daily. The case was seen twice a week for two months, after which the patient was discharged cured.

There seemed to be a molecular death of the cortical bone upon the exposed side, which the pyrozone removed. Upon the side of the cancellated bone vitality was maintained. The cortex became thinner and thinner, until granulations began to spring through the walls of the alveolus. No doubt there was some absorptive action upon the bone by the healthy tissues. Such action is not uncom-

---

[1] Dental Cosmos, vol. x. p. 635.

mon under other circumstances, as may be seen upon the roots of some chronically abscessed teeth, or upon planted teeth.

The foregoing treatment was merely experimental. The removal of the cortical bone by surgical means (*i.e.*, with a large bur in the engine) and the induction of tissue regeneration is preferable.

NECROSIS BEGINNING AT SOME HIGH PORTION OF THE ALVEOLUS, AS WHEN ARSENIC IS APPLIED THROUGH A PERFORATION.

The writer has seen several cases of this sort. When so applied, the necrosis extends in all directions, following the pericementum and attacking the bone. If promptly discovered and treated by removal of the dead portion, some hope may be entertained of retaining the tooth, but only at the expense of loss of tissue. If neglected, not only one but two teeth may be lost, this being dependent upon the quantity of arsenic applied.

Perforation by drilling is not always easy to differentiate from a partially devitalized pulp. In doubtful cases it is better to avoid the use of arsenic. The following is a description of the only case occurring in the writer's private practice:

One busy day a lady requested treatment of a left lower second bicuspid, which had been in the hands of an incompetent practitioner. The patient could give no history, except that much pain had been inflicted. Examination revealed what seemed to be a complete exposure of the pulp, but which results proved to be hypertrophied gum. The usual application was made, and the patient dismissed for a few days. Upon her return a necrosed condition of the septum between the second bicuspid and first molar was discovered. The bicuspid was opened and found to be perforated upon the distal face of the root. The opening was a line in diameter, and made evidently with the intention of opening the root-canal. The case was cured by extraction of the bicuspid and curetting the parts. The specimen is exhibited here, and it will be plain that any one would be warranted in following the treatment of the supposed pulp as was done.

In this connection it is well to call attention to the liability to this accident in cases of gum proliferation through a carious perforation of the root. The practice of establishing the diagnosis before attempting to apply arsenic is evidently correct.

NECROSIS BEGINNING AT THE APICAL FORAMEN.

There is no doubt in the writer's mind that many cases of chronic peridental irritation have occurred as the result of careless broaching. In all cases where paste has been applied, especially when placed in the root, the arsenic should be removed by alternate broaching and syringing. The writer has never been able to positively connect arsenic with apical irritation, but has suspected its occurrence after the manner indicated in a number of cases.

A unique case has been reported by a professional friend. He made an application to a pulp, but, finding it only partially devitalized thereby, he applied cocaine by cataphoresis without removing the traces of arsenical paste from the tooth.

A slough of three lines' diameter appeared over the apex, but was gradually thrown off by tissue regeneration beneath. Whether this result was due to cataphoric action or to mechanical conveyance of the arsenic to the apical space is not clear. This history may cause us to infer that there exists toleration of a certain amount of aseptic slough by the tissues of the apical space. We may bear in mind to what extent the escharotic action of zinc chloride and carbolic acid are tolerated by these parts.

### NECROSIS OF THE TISSUES OF THE CHEEK.

This simulates quite closely the ordinary aphthous ulcer, is usually slight, and requires little attention. If extensive, however, it should be treated upon the principle of exciting an inflammation to check the absorption of the drug. Free cauterization with argentic nitrate succeeded admirably in a case of deep penetration of arsenic into the tissues of the buccinator muscle at a point near the lower third molar. The treatment of arsenical necrosis of any magnitude with ferric hydrate has not resulted happily for the writer; perhaps the fault may lie in the difficulty of its maintenance in position. It is difficult to understand, however, why it should be expected to more than remove the unabsorbed arsenic from a part, inasmuch as dead tissue lies between it and the advancing arsenic.

It would be an omission, having stated the ill effects of arsenic when allowed to escape upon the gum, not to mention some means whereby leakage may be prevented. The quantity of paste used should be as minute as possible, and should be carefully sealed in the cavity. In accessible places, where the rubber dam can be applied, and especially in the lower teeth, the application may be made upon the under side of a small piece of letter-paper, and thin zinc phosphate flowed over it. The method is necessarily of limited application, owing to the unmanageable nature of the zinc phosphate. Temporary stopping is a material much lauded for this purpose. It should never be used in cavities the margins of which go beneath the gum, unless the rubber dam can be applied. As it requires a certain amount of pressure, which, if exerted upon the arsenic, will either insure its escape from the cavity or cause compression of the pulp, a cap of metal should be placed over the application. Pressure may then be exerted at will. Gutta-percha is a material too difficult of proper use to be relied upon in preference to temporary stopping.

A method employed by the writer is to first build in the cervical portion of the covering; next to make the application and cover it with a pellet of dry cotton; then to build in the remainder of the covering. Where cavity margins are in dangerous proximity to the gum, or where mechanical retention of the covering filling is but slight, and where the rubber dam cannot be applied, nothing can be better than an amalgam made from an alloy of forty parts silver, fifty-five parts tin, and five parts zinc, and known as "Flagg's facing." It absolutely does not leak, but probably expands, causes no pressure in its insertion, does not discolor within a considerable length of time, and is easily removed with an excavator. When desired, it may be relied upon for any reasonable period to maintain its position, even under the stress of mastication.

To illustrate: A right lower third molar had a large buccal cavity with the cervical margin under the gum, which also slightly overlapped it. The exposure of the pulp was complete, and business necessity rendered an immediate application desirable.

Though this represents, perhaps, an extreme case, yet the writer's experience is that in the clinical service frequently, and in private practice not so very seldom, cases of quite as puzzling a nature have to be dealt with. Facing amalgam, handled more or less in the manner about to be related, may be relied upon.

The cavity was first cleansed, and undercuts made along the mesial and distal walls. Disinfection with three per cent. hydrogen dioxide followed. A pellet of cotton was laid upon the pulp, and a small portion of soft amalgam was inserted back of the flap of gum and pressed outward, carrying the gum with it. The cavity was then filled with the amalgam. An excavator was now used to cut down through the buccal aspect of the filling upon the cotton, which was then removed. Arsenic upon cotton was placed upon the pulp, and a small portion of amalgam used to seal the tap. When it is desired to remove the covering, a dentated bur is passed into the tap, and the filling easily divided in two, when the halves may be crushed together. Ordinarily a small cervical portion may be left for the support of the clamp during treatment of the case. When this is completed it should, of course, be replaced by other material.

When the exposure is found upon a surface which is inaccessible by any ordinary method, as when a cervical cavity exists upon a distal face of a molar, what is known as a "pocket" should be made. This is a drill-pit made in the direction of the horn of the pulp, and preferably, for pathological reasons, towards one that is not exposed. Sometimes the pulp may be very closely approached. Arsenical paste is then sealed in the pocket, while soothing medicaments are to be placed in the cavity of decay. Of course, cataphoresis may be considered in any of these conditions, but with that we are not at present dealing.

Within the past few days, and since this article was written, the writer was consulted by a gentleman from Demarara, who desired some fillings made.

In the course of the operations mention was made of the excellent quality of his teeth, and sympathy expressed for the loss of every tooth back of the first bicuspid upon the right side of the lower jaw. He said he had with his fingers removed them all at once, together with the bone surrounding them, as the result of an application of "something to quiet an aching tooth." There was a very flat and greatly depressed condition of the ridge where the teeth had been lost, which seemed to bear out his statement. If there be truth in his story, we have no reason to doubt that arsenic was the medicament used, probably in considerable quantity. We may also learn to what extent necrosis may result without loss of a portion of the maxilla proper. The writer does not vouch for the case, but gives the facts as they were related to him. It may serve, at any rate, as a warning, and at the same time as an encouragement should an accident occur.

